# Progress in scaffold‐free bioprinting for cardiovascular medicine

**DOI:** 10.1111/jcmm.13598

**Published:** 2018-03-13

**Authors:** Nicanor I. Moldovan

**Affiliations:** ^1^ Departments of Biomedical Engineering and Ophthalmology 3D Bioprinting Core Indiana University‐Purdue University Indianapolis Indianapolis IN USA

**Keywords:** bioprinting, cardiovascular tissue engineering, cell spheroids, Kenzan method, scaffold‐free biofabrication

## Abstract

Biofabrication of tissue analogues is aspiring to become a disruptive technology capable to solve standing biomedical problems, from generation of improved tissue models for drug testing to alleviation of the shortage of organs for transplantation. Arguably, the most powerful tool of this revolution is bioprinting, understood as the assembling of cells with biomaterials in three‐dimensional structures. It is less appreciated, however, that bioprinting is not a uniform methodology, but comprises a variety of approaches. These can be broadly classified in two categories, based on the use or not of supporting biomaterials (known as “scaffolds,” usually printable hydrogels also called “bioinks”). Importantly, several limitations of scaffold‐dependent bioprinting can be avoided by the “scaffold‐free” methods. In this overview, we comparatively present these approaches and highlight the rapidly evolving scaffold‐free bioprinting, as applied to cardiovascular tissue engineering.

## 1 INTRODUCTION

There are several reasons to dedicate a special discussion to biofabrication and bioprinting for cardiovascular medicine. One is the intrinsic importance of the cardiovascular disorders, still the leading cause of mortality in United States and worldwide. One in three persons who recently died was of a cardiovascular disease in the United States only, and in other countries, the death toll was even higher.^1^ For this reason, some of the largest research efforts (and funding resources) have been traditionally put into this area. Moreover, the cardiovascular surgeons have been pioneering the direct tissue replacement by organ transplantation: this year is the 50th anniversary of the first heart transplantation by Christin Barnard in South Africa. Other approaches besides the pharmacological interventions aiming at structural repair and functional recovery are vascular grafting^2^ and the direct cell therapy. Therefore, it is no surprise that cardiovascular tissue engineering and, in particular, 3D printing and bioprinting are major areas of current interest and advances in cardiovascular medicine^3^.

Another reason of the importance bioprinting has for vascular biology is the universal need for vascularization of any tissue engineered construct larger than about half millimetre, as 200 μm is the limit of free oxygen diffusion in living tissues. Combined with the difficulty to provide an innervation, the lack of microvascular perfusion is a major roadblock to scaling‐up of many promising functional proofs of concept in biofabrication of live tissues.^4^

Here, we will leave out multiple applications of 3D printing (with no cells involved) to cardiovascular field, as well as the scaffold‐only printing,^5^ and the “hybrid” forms of bioprinting,^6^ which have been discussed in other excellent reviews recently.^3^ Instead, we will highlight scaffold‐free bioprinting as *bona fide* biofabrication, here understood as the creation of living, functional 3D constructs with cardiovascular applications.

## 2 BIOINK‐BASED BIOPRINTING AND ITS LIMITATIONS

As directly derived from 3D printing,^7^, ^8^ and thus inheriting much of the ongoing technological progress in additive manufacturing, one of the major advantages of the scaffold‐dependent bioprinting is the easiness to design and implement the construct's configuration, by direct image input via computer‐assisted design (CAD) files.^3^, ^7^ Another favourable feature, making it preferable for large, cell‐homogenous, matrix‐rich tissues such as bone, cartilage, muscle, is its excellent scalability.^9^ However, although new biological bioinks are emerging, for example, collagen‐ or fibrin‐based,^10^, ^11^ the structural cohesion (the “glue”) is obtained by still non‐universal, sometimes proprietary and/or expensive polymeric materials in form of hydrogels.^12^ As these hydrogels are essentially soft materials, in order to provide the constructs with the necessary biomechanical properties, they require a hardening step, usually a chemically‐ or UV‐induced polymerization.^13^ This can be cell‐damaging and thus significantly reduce the efficiency of the process. As an alternative is the “hybrid” bioprinting, consisting of incorporation in the construct of a second, usually fibrillary biomaterial.^6^

Cell loss may occur during material‐dependent bioprinting for a variety of method‐specific reasons. For example, during droplet generation substantial energy is delivered to the sample leading to vibration, heating and/or strong electrical fields.^12^ Milder bioprinting methods are currently developed, such as the laser‐assisted bioprinting.^14^ In this method, a laser pulse locally melts a “bioribbon” that consists of a cell‐embedding gel, thus generating a droplet deposited with high precision according to the desired 3D pattern. Still, cell viability could be an issue even with this method.^15^

Cells “encapsulation” within the supporting gel may additionally impair intercellular communication, although for several matrix‐rich tissue types such as bone and cartilage, this might be lesser of a problem. If the cells survive the initial isolation, this may be reduced in time with the slow diffusion of paracrine factors through the porous material and/or dissolution of the embedding matrix, followed by reducing of intercellular distances by proliferation and relocation.

As opposed to the surface which can be highly anatomically realistic, when examining the internal cellular architecture of most 3D constructs bioprinted so far,^16^, ^17^ their spatial organization is quite simplistic, following easily accessible geometric patterns rather than tissue structure, as tissues incorporate a larger amount of randomness and/or more structural refinement, such as fractal spatial distributions. 3D printing itself could be made ‐ in theory at least, given its high resolution ‐ more naturalistic; thus, this limitation might be considered to originate at the structural design stage. In some cases, after their initial deployment, the cells relocate within the bioprinted object, or following the biomaterial dissolution, allowing more natural cell arrangements.

Another essential issue facing biomaterial‐based bioprinting strategies is their biocompatibility. On the one hand, the energy‐intensive droplet producing processes may generate secondary molecular products from the gels, which could be directly cytotoxic either for the embedded cells,^18^ or for the recipient organism after construct's implantation. In addition, these biomaterials may trigger foreign‐body reactions to the implant.^7^ These will be likely less serious in the future when more “biological” bioinks will be used. Even so, both collagen and fibrin are reminiscent of wound‐healing and pro‐inflammatory processes, which might signal to the embedded cells subtle corresponding responses.

At the interface between scaffold‐dependent and scaffold‐free bioprinting lies the use of “bioinks” prepared exclusively from natural materials, such as collagen or fibrin, or even organ‐specific extracellular matrices.^19^ Although still experiencing some of the limitations of their deployment methods, the latter option is the most promising in terms of biocompatibility and capable of mitigating other downsides of the “bioinks” discussed above.

## 3 BIOMATERIAL‐INDEPENDENT (“SCAFFOLD‐FREE”) BIOPRINTING

Many of the problems related to the biomaterial use in bioprinting could be eliminated if only cells were used instead.^20^ One such problem is related to biocompatibility, which can be much increased, especially if patient‐derived cells are used (e.g., adult mesenchymal stem cells, or induced pluripotent stem cells, iPSC;^21^), possibly leading to creation of fully autologous constructs.^22^

Several such methods have been developed in the last couple of years, each with their advantages and disadvantages. A common limitation of this approach is that although cells can be manipulated individually, they do not easily form stable assemblies by simply bringing and maintaining them in contact, unless the intercellular adhesions are made very strong, possibly by chemical means.^23^ Additional structural cohesion needs to be produced by the cells, as their own secreted extracellular matrix. However, this may take longer time and depends on cell type and quality of matrix deposition. For this reason, the cells are first pre‐assembled in clusters (most often “spheroids”) and tested for the ability to secrete the “glue” needed to further combine them in larger‐scale constructs.^20^ Even so, the biomechanical properties of the constructs are less predictable than when using a pre‐defined material. If this matrix is insufficient or inappropriate for the contemplated applications, “hybrid” spheroids can be created, by adding supplementary matrix.^24^

The spheroid‐based methods are in general gentler and thus induce much less or no cell damage during the procedures. Another attractive feature of scaffold‐free bioprinting is its efficiency, as the speed can be comparable or even higher than other forms of bioprinting by using as “building blocks” large spheroids (in the tens of thousands cells^25^).

Although the scaffold‐free cell assembling methods do not have the common problems of inkjet and micro‐extrusion (such as the nozzle clogging), they still have their own technical limitations. One is the time of pre‐printing preparations which tend to be longer, while the post‐printing maturation time is probably comparable between the two approaches. Moreover, in such constructs, the cellular cross‐talk proceeds naturally, while optional addition of hydrogels in between the cells and within spheroids still remains possible and probably beneficial.^26^, ^27^ Thus, since scalability is more limited, scaffold‐free‐methods are preferable for smaller, cell‐heterogeneous, matrix‐poor tissues where the immediate (or continuous) intercellular communication is important.

A defining property of this biofabrication approach is that the tissue structure does not strictly follow a pre‐determined design, but emerges from fundamental developmental principles, more similar to embryological or organoid biology.^28^ The “voxel” of this form of bioprinting being the spheroid, a consequence is that the relative resolution is in the range of hundreds of microns. In practice, the positioning of cells can be known with much more precision because they attain predictable locations within the spheroids.^29^ Alternatively, more precision of cell location may not be needed, or could even be detrimental if it prevented the unfolding of self‐organization mechanisms during the so‐called post‐printing maturation phase.

Another implication of the basically biophysical nature of the factors at work during scaffold‐free biofabrication bears on the types of possible applications, as well as the required training, competencies and mindset of the users and of this technology's operators. Nevertheless, as a branch of bioengineering the scaffold‐free biofabrication remains a quantitative discipline, with the potential to benefit from advanced analytics and biosensors, molecular‐level optimization and computer modelling.^30^, ^31^, ^32^

## 4 HYDROGELS AS TEMPORARY SCAFFOLDS

Steps towards cells‐only bioprinting were previously attained by using “sacrificial” or “fugitive” hydrogels. For example, one of the best‐known bioprinting company in United States is Organovo, which launched the bioprinter NovoGen. On this machine, pre‐formed cylindrical cell aggregates are placed in between alginate rods of similar diameters in pre‐defined geometry and held in place until the cellular structures fuse in a continuous cellular mass. Then, the hydrogel is dissolved, leaving behind a “scaffold‐free” construct. By this approach, vascular,^33^ nerve,^34^ liver^35^ and kidney^36^ structures were demonstrated and now commercialized for in vitro pharmacological assays.

Another technology is that of the company 3D Bioprinting Solutions, which similarly employs “fugitive” hydrogels for holding in place cells spheroids for fusion in larger‐scale structures ‐ in this case, thyroid glands, currently being tested in athymic mice.^37^ Similarly, this approach of high‐density cell cords was adapted to bioprinting of vascular tubes from cells compacted in alginate tubes and then re‐loaded after supporting hydrogel removal in an extrusion bioprinter for 3D assembling.^38^ In all these cases, at least at one point in the process the cells are placed in contact with the biomaterial, with all the implications discussed before.

Cell‐sheet technology, depending on a detachable polymeric substrate for cellular sheet lifting, can also be used to create more sophisticated 3D structures.^39^

## 5 RECENT DEVELOPMENTS IN CARDIOVASCULAR SCAFFOLD‐FREE 3D BIOPRINTING

A variety of hydrogel‐free methods to make 3D tissue analogues also exist. For space limitations, we'll address only two versions, magnetic and mechanical (microneedles) methods.

### 5.1 Magnetic assembling of model cardiovascular tissues

In theory at least, the cells can be brought together in 3D structures and maintained in position for long enough to interact and reconstitute their native environment, using the magnetic force. Magnetic micro‐ and nano‐beads are creatively and intensively explored for a variety of applications following this paradigm.

In one of its embodiments, the magnetic method speeds up the formation of spheroids and then their assembling into larger constructs. Two groups are particularly active in this area and thus deserve special attention. One is the company Nano3D, which commercializes a kit allowing the labelling of cells with nano‐particles and the magnet to pull them down (“bioprint”) or up (“levitate”).^40^ The beads bind the cell surface non‐specifically (via electrostatic forces) and reversibly, as they are eventually either released in the medium or internalized to some extent (without detectable side‐effects on the cells). Therefore, the method is said to be universal regarding the phenotype of the involved cells, although the stability of the spheroids and thus of the resulting constructs may vary. Using this regent, a series of high‐profile papers have been published, with examples in vascular,^41^, ^42^ pulmonary^43^ and tumour,^44^, ^45^ biology.

Other similar magnetic beads‐based methods are pursued in academic environments. Relevant to this discussion are the Janus'‐like microbeads which are internalized^46^ and were demonstrated to assist the formation of vascular‐relevant structures.^47^, ^48^, ^49^ While these methods are definitely useful for in vitro models and experimentation, their translation to clinical practice is more questionable, particularly due to the limited size of the constructs and the residual magnetic material.

### 5.2 The microneedles‐based “Kenzan” bioprinting

**Figure 1:**
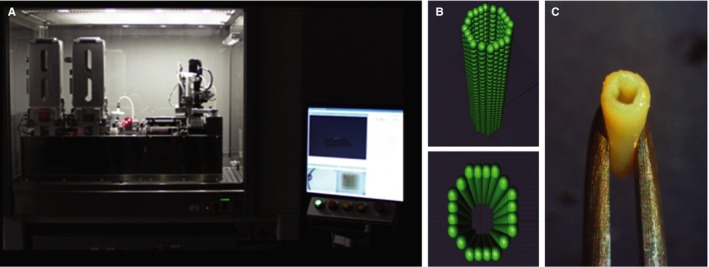
Scaffold‐free bioprinting of a vascular graft on the Regenova bioprinter. A, Frontal view of the robot, together with its controlling computer. B, Virtual design of the spheroids positioning in the tube. C, Actual construct demonstrating surgical robustness for implantation (*modified with permission from Itoh et al* 51)

A radical alternative to hydrogels for providing a temporary support to spheroids, and thus facilitating their fusion and maturation in meaningful tissue models, is the use of a set of microneedles (“Kenzan” in Japanese).^50^ This method is implemented in the Regenova “Bio‐3D Printer” commercialized by Cyfuse Biomedical K.K. and in United States by its subsidiary Amuza, Inc. (Figure 1A). Currently, there are several such instruments operational in Japan and a few in the United States (to our knowledge, two in academic and one in a corporate institution). For a comprehensive discussion of this technology, we suggest our recent review.^25^

Several functional tissue constructs have been assembled so far on the Regenova robot. Among them is a small‐diameter (inner diameter of 1.5 mm and 7 mm in length) vascular graft tested in vivo.^51^ To this end, human cell spheroids were prepared from 2.5 × 10^4^ cells/spheroid of 40% umbilical vein endothelial cells (EC), 10% aortic smooth muscle cells and 50% dermal fibroblasts (FB) and maintained in a cocktail of corresponding growth media in the proportion 1:1:1. These spheroids were printed as tubular constructs in the microneedles following a pre‐designed pattern (Figure 1B) and maintained for fusion for 4 days. Then, the tubes were removed (Figure 1C) and perfused for an additional 7 days. The tubes were surgically implanted and sutured into the abdominal aortas of nude rats. Blood flow was assessed the by ultrasonography, and all grafts were found to be patent for the 5 days of the experiment. This was terminated due to significant enlargement and thinning of the grafts. At immuno‐histological examination, EC were found redistributed to an intimal layer, probably explaining the patency of these grafts.

**Figure 2:**
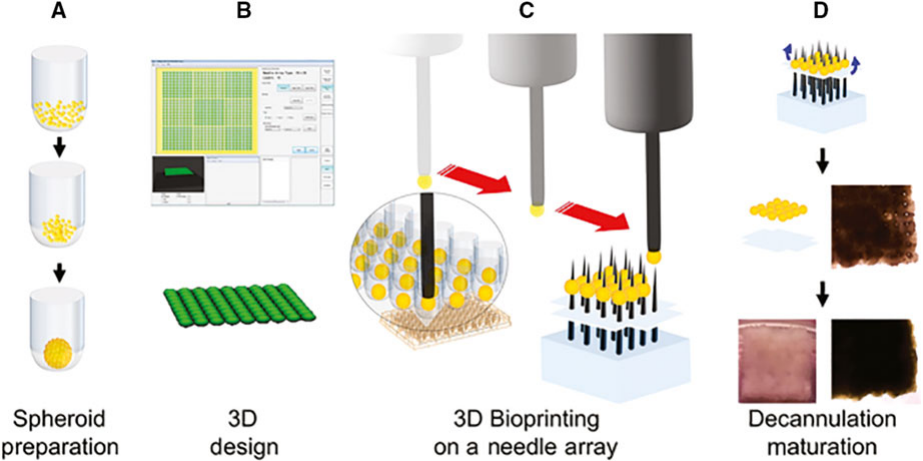
Schematic of biomaterial‐free bioprinting of a cardiac patch. A, Cells (CM, FB, EC) are aggregated in ultra‐low attachment 96‐well plates to form spheroids. B, The desired 3D structure is designed using computer software. C, The robot picks up individual spheroids using vacuum suction and loads them onto a needle array. D, Spheroids are allowed to fuse. The 3D bioprinted cardiac tissue is then removed from the needle array and further cultured to allow the needle holes to be resorbed (*reproduced with permission from Ong et al* 56)

Using the same technology, tracheal^52^ and uretral^53^ tubes were obtained, as well as neural bridges^54^ and liver buds.^55^ Yet probably the most notable accomplishment to date is the bioprinting of a functional cardiac patch^56^ (Figure 2). The spheroids were prepared from human induced pluripotent stem cell‐derived cardiomyocytes (CM), FB and EC, in the proportions CM:FB:EC = 70:15:15, 70:0:30 or 45:40:15. These were assembled using the Regenova bioprinter. After bioprinting, these cardiac tissue patches of all cell ratios not only beat spontaneously, but also exhibited ventricular‐like action potential waveforms and uniform electrical conduction. Reproducing a feature of cardiac fibrosis, the conduction velocities were higher and action potential durations were longer in patches containing a lower percentage of FB. CM, FB and EC markers were detected by immunohistochemistry both in spheroids and in the formatted tissue patches. EC displayed signs of self‐assembling towards capillary primordia. After implantation on the surface of the myocardium in immune‐deficient rats, these pre‐vascular structures became perfused with recipient blood, indicating spontaneous anastomosis.

In conclusion, cardiovascular tissue engineering benefits from recent technological progress, in particular from bioprinting, which comes in two complementary flavours, as dependent or not on a bioink. Collectively, both these approaches hold the potential to transform the way cardiovascular diseases are treated in the future (see Table 1 for a summary comparison of scaffold‐dependent and independent methods).

**Table 1:** Comparative features and some advantages of scaffold‐free biofabrication. For details, see text

Scaffold‐dependent bioprinting	Scaffold‐free biofabrication
Bioinks are essentially soft biomaterials	Cells produce optimal matrix
Hardening is non‐trivial and consequential	No stiffness adjustment necessary
A “universal” bioink is yet to be found	Biomaterials can still be optionally added
Limited intercellular communication	Natural intercellular interactions
Variable cell damage	Higher efficiency, less cell damage
Biocompatibility more difficult to attain	Easier obtained biocompatibility
Simplistic cellular architecture	Closer following of developmental principles
Good for large, cell‐homogenous, matrix‐rich tissue	Best for smaller, cell‐heterogeneous, matrix‐poor tissues

## CONFLICT OF INTERESTS

The author confirms that there are no conflicts of interest.

## AUTHOR CONTRIBUTION

NM conceived and wrote the manuscript.
